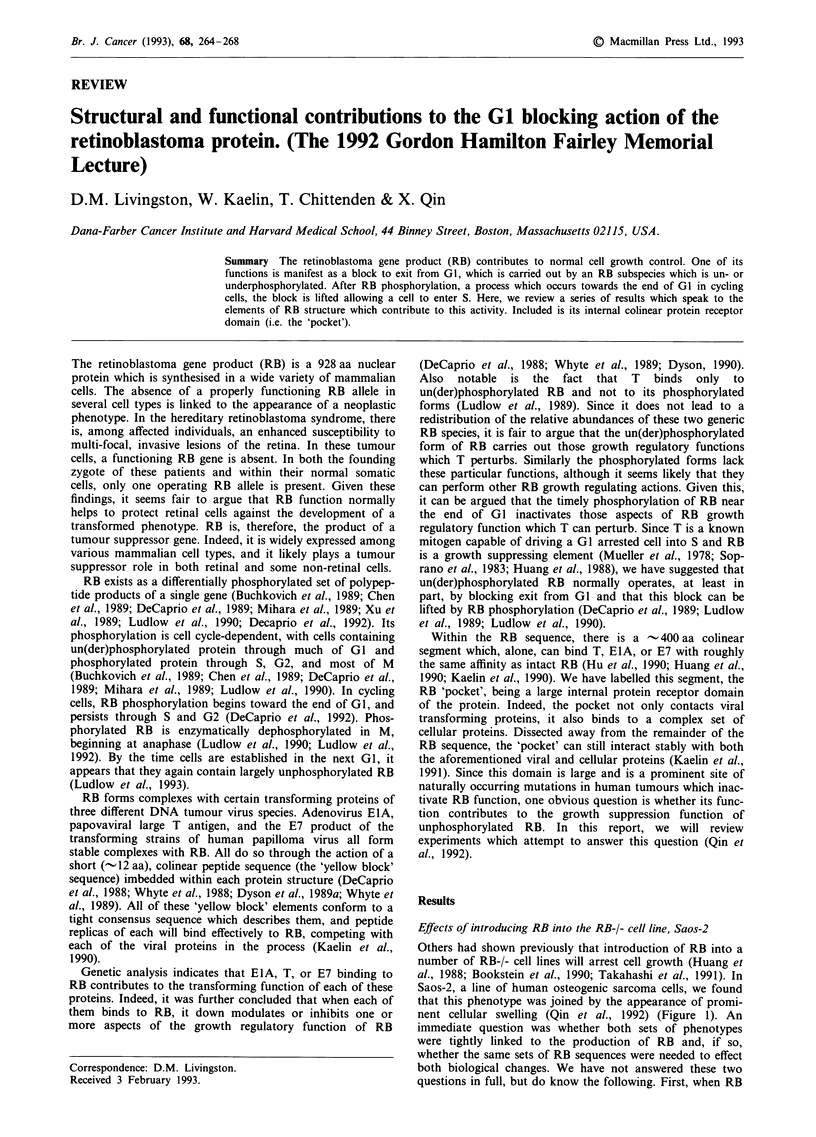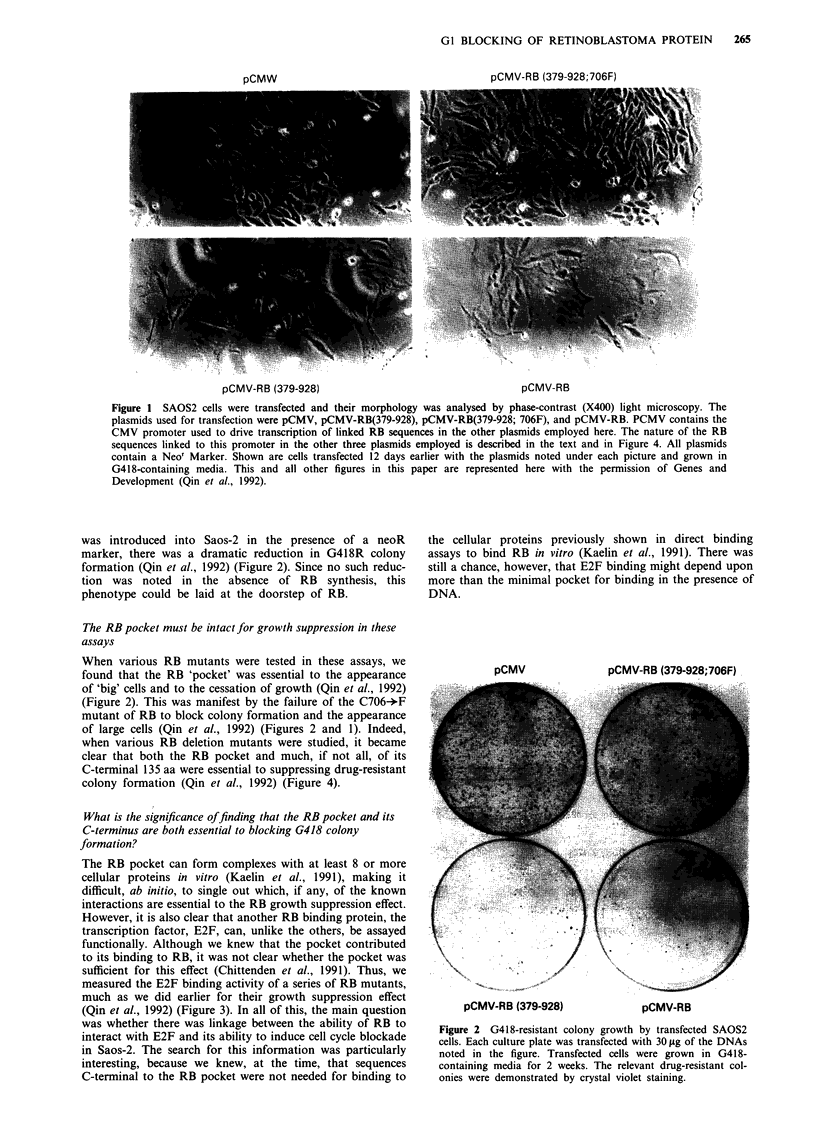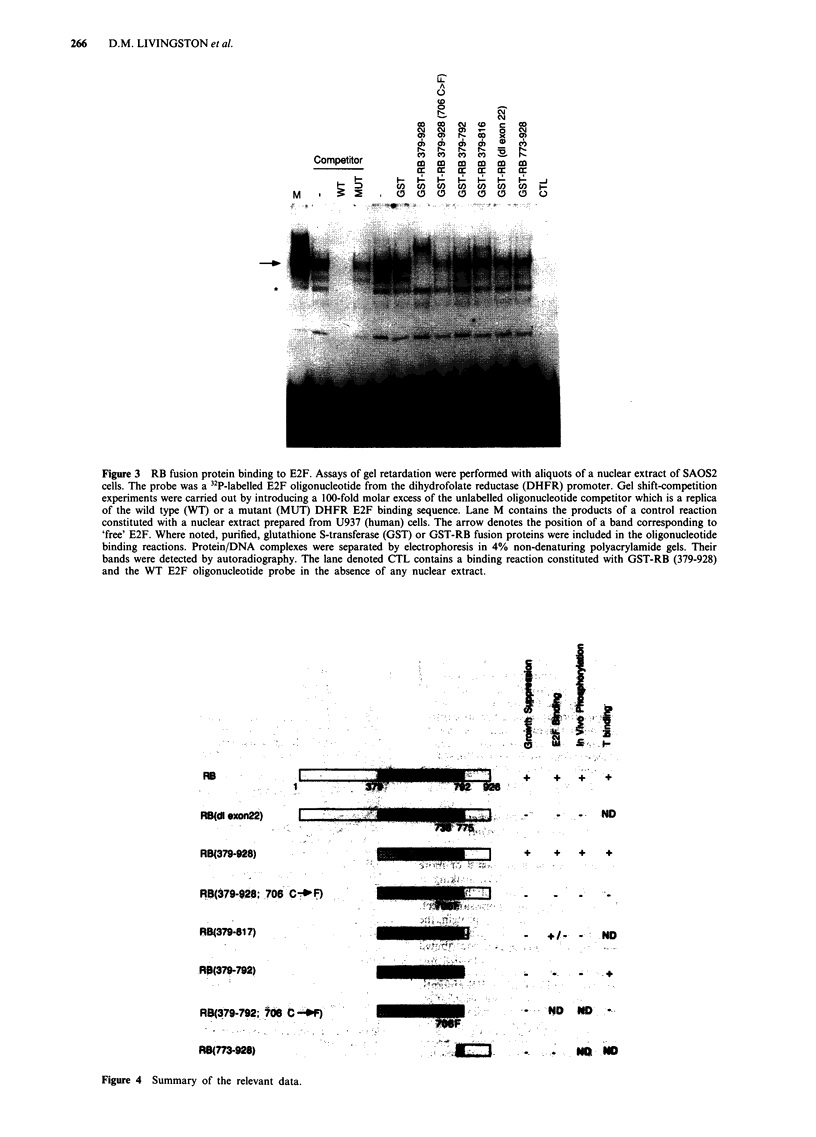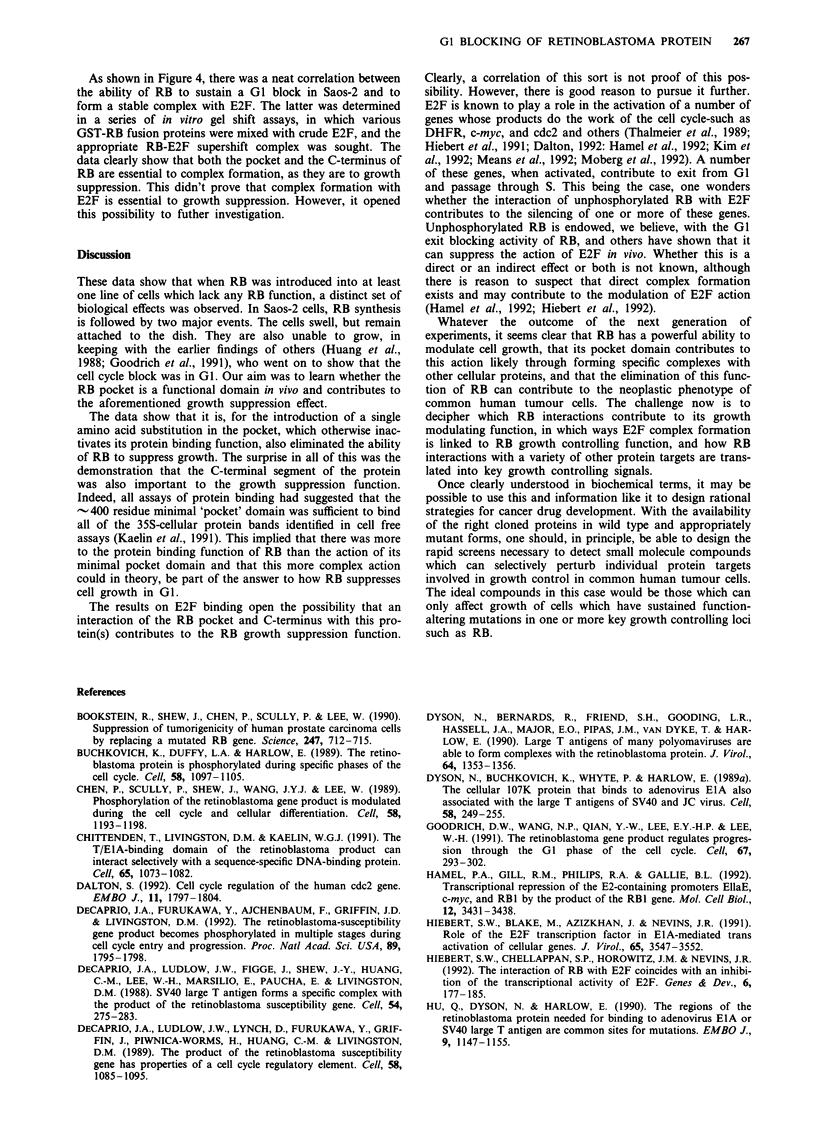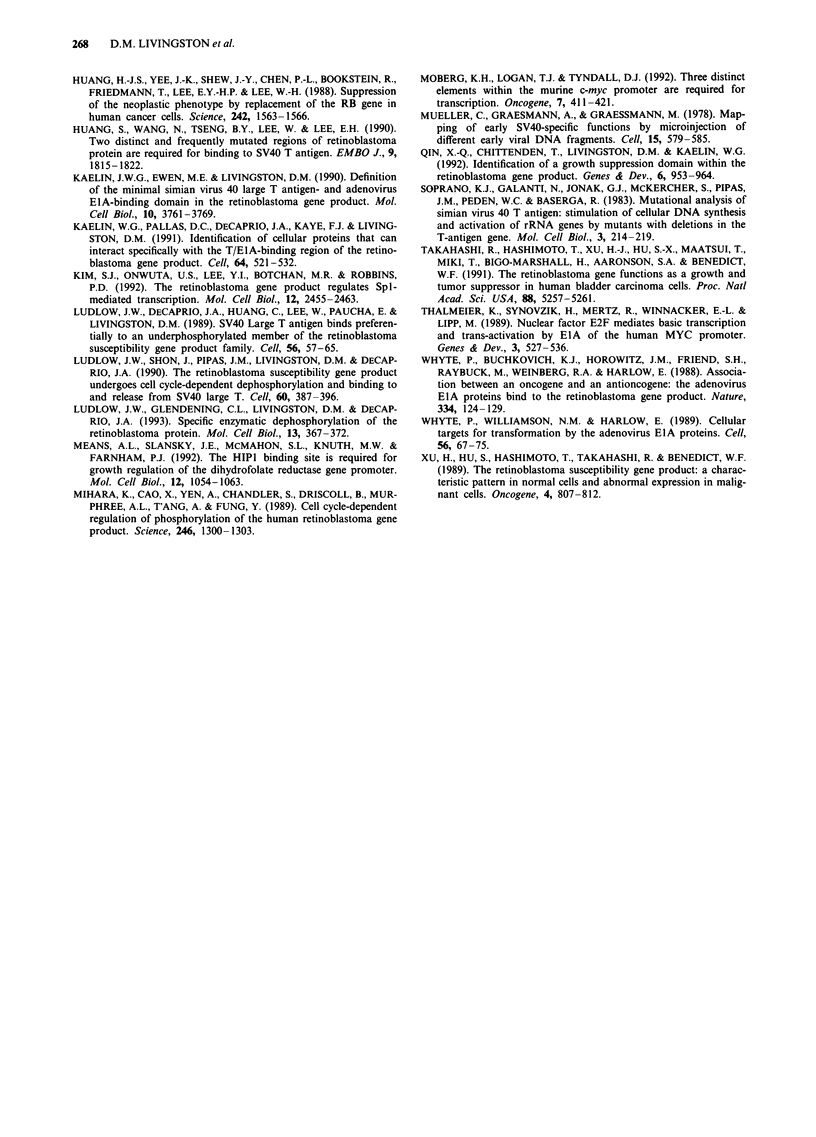# Structural and functional contributions to the G1 blocking action of the retinoblastoma protein. (the 1992 Gordon Hamilton Fairley Memorial Lecture).

**DOI:** 10.1038/bjc.1993.325

**Published:** 1993-08

**Authors:** D. M. Livingston, W. Kaelin, T. Chittenden, X. Qin

**Affiliations:** Dana-Farber Cancer Institute, Boston, Massachusetts.

## Abstract

**Images:**


*Br. J. Cancer* (1993), **68**, 264–268 © Macmillan Press Ltd., 1993

**REVIEW**

### Structural and functional contributions to the G1 blocking action of the retinoblastoma protein. (The 1992 Gordon Hamilton Fairley Memorial Lecture)

D.M. Livingston, W. Kaelin, T. Chittenden & X. Qin

*Dana-Farber Cancer Institute and Harvard Medical School, 44 Binney Street, Boston, Massachusetts 02115, USA.*

**Summary** The retinoblastoma gene product (RB) contributes to normal cell growth control. One of its functions is manifest as a block to exit from G1, which is carried out by an RB subspecies which is un- or underphosphorylated. After RB phosphorylation, a process which occurs towards the end of G1 in cycling cells, the block is lifted allowing a cell to enter S. Here, we review a series of results which speak to the elements of RB structure which contribute to this activity. Included is its internal colinear protein receptor domain (i.e. the 'pocket').

The retinoblastoma gene product (RB) is a 928 aa nuclear protein which is synthesised in a wide variety of mammalian cells. The absence of a properly functioning RB allele in several cell types is linked to the appearance of a neoplastic phenotype. In the hereditary retinoblastoma syndrome, there is, among affected individuals, an enhanced susceptibility to multi-focal, invasive lesions of the retina. In these tumour cells, a functioning RB gene is absent. In both the founding zygote of these patients and within their normal somatic cells, only one operating RB allele is present. Given these findings, it seems fair to argue that RB function normally helps to protect retinal cells against the development of a transformed phenotype. RB is, therefore, the product of a tumour suppressor gene. Indeed, it is widely expressed among various mammalian cell types, and it likely plays a tumour suppressor role in both retinal and some non-retinal cells.

RB exists as a differentially phosphorylated set of polypeptide products of a single gene (Buchkovich *et al.*, 1989; Chen *et al.*, 1989; DeCaprio *et al.*, 1989; Mihara *et al.*, 1989; Xu *et al.*, 1989; Ludlow *et al.*, 1990; Decaprio *et al.*, 1992). Its phosphorylation is cell cycle-dependent, with cells containing un(der)phosphorylated protein through much of G1 and phosphorylated protein through S, G2, and most of M (Buchkovich *et al.*, 1989; Chen *et al.*, 1989; DeCaprio *et al.*, 1989; Mihara *et al.*, 1989; Ludlow *et al.*, 1990). In cycling cells, RB phosphorylation begins toward the end of G1, and persists through S and G2 (DeCaprio *et al.*, 1992). Phosphorylated RB is enzymatically dephosphorylated in M, beginning at anaphase (Ludlow *et al.*, 1990; Ludlow *et al.*, 1992). By the time cells are established in the next G1, it appears that they again contain largely unphosphorylated RB (Ludlow *et al.*, 1993).

RB forms complexes with certain transforming proteins of three different DNA tumour virus species. Adenovirus E1A, papovaviral large T antigen, and the E7 product of the transforming strains of human papilloma virus all form stable complexes with RB. All do so through the action of a short (~12 aa), colinear peptide sequence (the 'yellow block' sequence) imbedded within each protein structure (DeCaprio *et al.*, 1988; Whyte *et al.*, 1988; Dyson *et al.*, 1989*a*; Whyte *et al.*, 1989). All of these 'yellow block' elements conform to a tight consensus sequence which describes them, and peptide replicas of each will bind effectively to RB, competing with each of the viral proteins in the process (Kaelin *et al.*, 1990).

Genetic analysis indicates that E1A, T, or E7 binding to RB contributes to the transforming function of each of these proteins. Indeed, it was further concluded that when each of them binds to RB, it down modulates or inhibits one or more aspects of the growth regulatory function of RB (DeCaprio *et al.*, 1988; Whyte *et al.*, 1989; Dyson, 1990). Also notable is the fact that T binds only to un(der)phosphorylated RB and not to its phosphorylated forms (Ludlow *et al.*, 1989). Since it does not lead to a redistribution of the relative abundances of these two generic RB species, it is fair to argue that the un(der)phosphorylated form of RB carries out those growth regulatory functions which T perturbs. Similarly the phosphorylated forms lack these particular functions, although it seems likely that they can perform other RB growth regulating actions. Given this, it can be argued that the timely phosphorylation of RB near the end of G1 inactivates those aspects of RB growth regulatory function which T can perturb. Since T is a known mitogen capable of driving a G1 arrested cell into S and RB is a growth suppressing element (Mueller *et al.*, 1978; Soprano *et al.*, 1983; Huang *et al.*, 1988), we have suggested that un(der)phosphorylated RB normally operates, at least in part, by blocking exit from G1 and that this block can be lifted by RB phosphorylation (DeCaprio *et al.*, 1989; Ludlow *et al.*, 1989; Ludlow *et al.*, 1990).

Within the RB sequence, there is a ~400 aa colinear segment which, alone, can bind T, E1A, or E7 with roughly the same affinity as intact RB (Hu *et al.*, 1990; Huang *et al.*, 1990; Kaelin *et al.*, 1990). We have labelled this segment, the RB 'pocket', being a large internal protein receptor domain of the protein. Indeed, the pocket not only contacts viral transforming proteins, it also binds to a complex set of cellular proteins. Dissected away from the remainder of the RB sequence, the 'pocket' can still interact stably with both the aforementioned viral and cellular proteins (Kaelin *et al.*, 1991). Since this domain is large and is a prominent site of naturally occurring mutations in human tumours which inactivate RB function, one obvious question is whether its function contributes to the growth suppression function of unphosphorylated RB. In this report, we will review experiments which attempt to answer this question (Qin *et al.*, 1992).

### Results

### *Effects of introducing RB into the RB-/- cell line, Saos-2*

Others had shown previously that introduction of RB into a number of RB-/- cell lines will arrest cell growth (Huang *et al.*, 1988; Bookstein *et al.*, 1990; Takahashi *et al.*, 1991). In Saos-2, a line of human osteogenic sarcoma cells, we found that this phenotype was joined by the appearance of prominent cellular swelling (Qin *et al.*, 1992) (Figure 1). An immediate question was whether both sets of phenotypes were tightly linked to the production of RB and, if so, whether the same sets of RB sequences were needed to effect both biological changes. We have not answered these two questions in full, but do know the following. First, when RB

Correspondence: D.M. Livingston.
Received 3 February 1993.

**Figure 1** SAOS2 cells were transfected and their morphology was analysed by phase-contrast (X400) light microscopy. The plasmids used for transfection were pCMV, pCMV-RB(379-928), pCMV-RB(379-928; 706F), and pCMV-RB. PCMV contains the CMV promoter used to drive transcription of linked RB sequences in the other plasmids employed here. The nature of the RB sequences linked to this promoter in the other three plasmids employed is described in the text and in Figure 4. All plasmids contain a Neo$^r$ Marker. Shown are cells transfected 12 days earlier with the plasmids noted under each picture and grown in G418-containing media. This and all other figures in this paper are represented here with the permission of Genes and Development (Qin *et al.*, 1992).

was introduced into Saos-2 in the presence of a neoR marker, there was a dramatic reduction in G418R colony formation (Qin *et al.*, 1992) (Figure 2). Since no such reduction was noted in the absence of RB synthesis, this phenotype could be laid at the doorstep of RB.

*The RB pocket must be intact for growth suppression in these assays*

When various RB mutants were tested in these assays, we found that the RB 'pocket' was essential to the appearance of 'big' cells and to the cessation of growth (Qin *et al.*, 1992) (Figure 2). This was manifest by the failure of the C706→F mutant of RB to block colony formation and the appearance of large cells (Qin *et al.*, 1992) (Figures 2 and 1). Indeed, when various RB deletion mutants were studied, it became clear that both the RB pocket and much, if not all, of its C-terminal 135 aa were essential to suppressing drug-resistant colony formation (Qin *et al.*, 1992) (Figure 4).

*What is the significance of finding that the RB pocket and its C-terminus are both essential to blocking G418 colony formation?*

The RB pocket can form complexes with at least 8 or more cellular proteins *in vitro* (Kaelin *et al.*, 1991), making it difficult, *ab initio*, to single out which, if any, of the known interactions are essential to the RB growth suppression effect. However, it is also clear that another RB binding protein, the transcription factor, E2F, can, unlike the others, be assayed functionally. Although we knew that the pocket contributed to its binding to RB, it was not clear whether the pocket was sufficient for this effect (Chittenden *et al.*, 1991). Thus, we measured the E2F binding activity of a series of RB mutants, much as we did earlier for their growth suppression effect (Qin *et al.*, 1992) (Figure 3). In all of this, the main question was whether there was linkage between the ability of RB to interact with E2F and its ability to induce cell cycle blockade in Saos-2. The search for this information was particularly interesting, because we knew, at the time, that sequences C-terminal to the RB pocket were not needed for binding to the cellular proteins previously shown in direct binding assays to bind RB *in vitro* (Kaelin *et al.*, 1991). There was still a chance, however, that E2F binding might depend upon more than the minimal pocket for binding in the presence of DNA.

**Figure 2** G418-resistant colony growth by transfected SAOS2 cells. Each culture plate was transfected with 30 μg of the DNAs noted in the figure. Transfected cells were grown in G418-containing media for 2 weeks. The relevant drug-resistant colonies were demonstrated by crystal violet staining.

**Figure 3** RB fusion protein binding to E2F. Assays of gel retardation were performed with aliquots of a nuclear extract of SAOS2 cells. The probe was a $^{32}$P-labelled E2F oligonucleotide from the dihydrofolate reductase (DHFR) promoter. Gel shift-competition experiments were carried out by introducing a 100-fold molar excess of the unlabelled oligonucleotide competitor which is a replica of the wild type (WT) or a mutant (MUT) DHFR E2F binding sequence. Lane M contains the products of a control reaction constituted with a nuclear extract prepared from U937 (human) cells. The arrow denotes the position of a band corresponding to 'free' E2F. Where noted, purified, glutathione S-transferase (GST) or GST-RB fusion proteins were included in the oligonucleotide binding reactions. Protein/DNA complexes were separated by electrophoresis in 4% non-denaturing polyacrylamide gels. Their bands were detected by autoradiography. The lane denoted CTL contains a binding reaction constituted with GST-RB (379-928) and the WT E2F oligonucleotide probe in the absence of any nuclear extract.

**Figure 4** Summary of the relevant data.

As shown in Figure 4, there was a neat correlation between the ability of RB to sustain a G1 block in Saos-2 and to form a stable complex with E2F. The latter was determined in a series of *in vitro* gel shift assays, in which various GST-RB fusion proteins were mixed with crude E2F, and the appropriate RB-E2F supershift complex was sought. The data clearly show that both the pocket and the C-terminus of RB are essential to complex formation, as they are to growth suppression. This didn't prove that complex formation with E2F is essential to growth suppression. However, it opened this possibility to futher investigation.

### Discussion

These data show that when RB was introduced into at least one line of cells which lack any RB function, a distinct set of biological effects was observed. In Saos-2 cells, RB synthesis is followed by two major events. The cells swell, but remain attached to the dish. They are also unable to grow, in keeping with the earlier findings of others (Huang *et al.*, 1988; Goodrich *et al.*, 1991), who went on to show that the cell cycle block was in G1. Our aim was to learn whether the RB pocket is a functional domain *in vivo* and contributes to the aforementioned growth suppression effect.

The data show that it is, for the introduction of a single amino acid substitution in the pocket, which otherwise inactivates its protein binding function, also eliminated the ability of RB to suppress growth. The surprise in all of this was the demonstration that the C-terminal segment of the protein was also important to the growth suppression function. Indeed, all assays of protein binding had suggested that the ~400 residue minimal 'pocket' domain was sufficient to bind all of the 35S-cellular protein bands identified in cell free assays (Kaelin *et al.*, 1991). This implied that there was more to the protein binding function of RB than the action of its minimal pocket domain and that this more complex action could in theory, be part of the answer to how RB suppresses cell growth in G1.

The results on E2F binding open the possibility that an interaction of the RB pocket and C-terminus with this protein(s) contributes to the RB growth suppression function. Clearly, a correlation of this sort is not proof of this possibility. However, there is good reason to pursue it further. E2F is known to play a role in the activation of a number of genes whose products do the work of the cell cycle-such as DHFR, c-*myc*, and cdc2 and others (Thalmeier *et al.*, 1989; Hiebert *et al.*, 1991; Dalton, 1992: Hamel *et al.*, 1992; Kim *et al.*, 1992; Means *et al.*, 1992; Moberg *et al.*, 1992). A number of these genes, when activated, contribute to exit from G1 and passage through S. This being the case, one wonders whether the interaction of unphosphorylated RB with E2F contributes to the silencing of one or more of these genes. Unphosphorylated RB is endowed, we believe, with the G1 exit blocking activity of RB, and others have shown that it can suppress the action of E2F *in vivo*. Whether this is a direct or an indirect effect or both is not known, although there is reason to suspect that direct complex formation exists and may contribute to the modulation of E2F action (Hamel *et al.*, 1992; Hiebert *et al.*, 1992).

Whatever the outcome of the next generation of experiments, it seems clear that RB has a powerful ability to modulate cell growth, that its pocket domain contributes to this action likely through forming specific complexes with other cellular proteins, and that the elimination of this function of RB can contribute to the neoplastic phenotype of common human tumour cells. The challenge now is to decipher which RB interactions contribute to its growth modulating function, in which ways E2F complex formation is linked to RB growth controlling function, and how RB interactions with a variety of other protein targets are translated into key growth controlling signals.

Once clearly understood in biochemical terms, it may be possible to use this and information like it to design rational strategies for cancer drug development. With the availability of the right cloned proteins in wild type and appropriately mutant forms, one should, in principle, be able to design the rapid screens necessary to detect small molecule compounds which can selectively perturb individual protein targets involved in growth control in common human tumour cells. The ideal compounds in this case would be those which can only affect growth of cells which have sustained function-altering mutations in one or more key growth controlling loci such as RB.

### References

BOOKSTEIN, R., SHEW, J., CHEN, P., SCULLY, P. & LEE, W. (1990). Suppression of tumorigenicity of human prostate carcinoma cells by replacing a mutated RB gene. *Science*, **247**, 712–715.

BUCHKOVICH, K., DUFFY, L.A. & HARLOW, E. (1989). The retinoblastoma protein is phosphorylated during specific phases of the cell cycle. *Cell*, **58**, 1097–1105.

CHEN, P., SCULLY, P., SHEW, J., WANG, J.Y.J. & LEE, W. (1989). Phosphorylation of the retinoblastoma gene product is modulated during the cell cycle and cellular differentiation. *Cell*, **58**, 1193–1198.

CHITTENDEN, T., LIVINGSTON, D.M. & KAELIN, W.G.J. (1991). The T/E1A-binding domain of the retinoblastoma product can interact selectively with a sequence-specific DNA-binding protein. *Cell*, **65**, 1073–1082.

DALTON, S. (1992). Cell cycle regulation of the human cdc2 gene. *EMBO J.*, **11**, 1797–1804.

DECAPRIO, J.A., FURUKAWA, Y., AJCHENBAUM, F., GRIFFIN, J.D. & LIVINGSTON, D.M. (1992). The retinoblastoma-susceptibility gene product becomes phosphorylated in multiple stages during cell cycle entry and progression. *Proc. Natl Acad. Sci. USA*, **89**, 1795–1798.

DECAPRIO, J.A., LUDLOW, J.W., FIGGE, J., SHEW, J.-Y., HUANG, C.-M., LEE, W.-H., MARSILIO, E., PAUCHA, E. & LIVINGSTON, D.M. (1988). SV40 large T antigen forms a specific complex with the product of the retinoblastoma susceptibility gene. *Cell*, **54**, 275–283.

DECAPRIO, J.A., LUDLOW, J.W., LYNCH, D., FURUKAWA, Y., GRIFFIN, J., PIWNICA-WORMS, H., HUANG, C.-M. & LIVINGSTON, D.M. (1989). The product of the retinoblastoma susceptibility gene has properties of a cell cycle regulatory element. *Cell*, **58**, 1085–1095.

DYSON, N., BERNARDS, R., FRIEND, S.H., GOODING, L.R., HASSELL, J.A., MAJOR, E.O., PIPAS, J.M., VAN DYKE, T. & HARLOW, E. (1990). Large T antigens of many polyomaviruses are able to form complexes with the retinoblastoma protein. *J. Virol.*, **64**, 1353–1356.

DYSON, N., BUCHKOVICH, K., WHYTE, P. & HARLOW, E. (1989*a*). The cellular 107K protein that binds to adenovirus E1A also associated with the large T antigens of SV40 and JC virus. *Cell*, **58**, 249–255.

GOODRICH, D.W., WANG, N.P., QIAN, Y.-W., LEE, E.Y.-H.P. & LEE, W.-H. (1991). The retinoblastoma gene product regulates progression through the G1 phase of the cell cycle. *Cell*, **67**, 293–302.

HAMEL, P.A., GILL, R.M., PHILIPS, R.A. & GALLIE, B.L. (1992). Transcriptional repression of the E2-containing promoters EIIaE, c-*myc*, and RB1 by the product of the RB1 gene. *Mol. Cell Biol.*, **12**, 3431–3438.

HIEBERT, S.W., BLAKE, M., AZIZKHAN, J. & NEVINS, J.R. (1991). Role of the E2F transcription factor in E1A-mediated trans activation of cellular genes. *J. Virol.*, **65**, 3547–3552.

HIEBERT, S.W., CHELLAPPAN, S.P., HOROWITZ, J.M. & NEVINS, J.R. (1992). The interaction of RB with E2F coincides with an inhibition of the transcriptional activity of E2F. *Genes & Dev.*, **6**, 177–185.

HU, Q., DYSON, N. & HARLOW, E. (1990). The regions of the retinoblastoma protein needed for binding to adenovirus E1A or SV40 large T antigen are common sites for mutations. *EMBO J.*, **9**, 1147–1155.

HUANG, H.-J.S., YEE, J.-K., SHEW, J.-Y., CHEN, P.-L., BOOKSTEIN, R., FRIEDMANN, T., LEE, E.Y.-H.P. & LEE, W.-H. (1988). Suppression of the neoplastic phenotype by replacement of the RB gene in human cancer cells. *Science*, **242**, 1563–1566.

HUANG, S., WANG, N., TSENG, B.Y., LEE, W. & LEE, E.H. (1990). Two distinct and frequently mutated regions of retinoblastoma protein are required for binding to SV40 T antigen. *EMBO J.*, **9**, 1815–1822.

KAELIN, J.W.G., EWEN, M.E. & LIVINGSTON, D.M. (1990). Definition of the minimal simian virus 40 large T antigen- and adenovirus E1A-binding domain in the retinoblastoma gene product. *Mol. Cell Biol.*, **10**, 3761–3769.

KAELIN, W.G., PALLAS, D.C., DECAPRIO, J.A., KAYE, F.J. & LIVINGSTON, D.M. (1991). Identification of cellular proteins that can interact specifically with the T/E1A-binding region of the retinoblastoma gene product. *Cell*, **64**, 521–532.

KIM, S.J., ONWUTA, U.S., LEE, Y.I., BOTCHAN, M.R. & ROBBINS, P.D. (1992). The retinoblastoma gene product regulates Sp1-mediated transcription. *Mol. Cell Biol.*, **12**, 2455–2463.

LUDLOW, J.W., DECAPRIO, J.A., HUANG, C., LEE, W., PAUCHA, E. & LIVINGSTON, D.M. (1989). SV40 Large T antigen binds preferentially to an underphosphorylated member of the retinoblastoma susceptibility gene product family. *Cell*, **56**, 57–65.

LUDLOW, J.W., SHON, J., PIPAS, J.M., LIVINGSTON, D.M. & DECAPRIO, J.A. (1990). The retinoblastoma susceptibility gene product undergoes cell cycle-dependent dephosphorylation and binding to and release from SV40 large T. *Cell*, **60**, 387–396.

LUDLOW, J.W., GLENDENING, C.L., LIVINGSTON, D.M. & DECAPRIO, J.A. (1993). Specific enzymatic dephosphorylation of the retinoblastoma protein. *Mol. Cell Biol.*, **13**, 367–372.

MEANS, A.L., SLANSKY, J.E., MCMAHON, S.L., KNUTH, M.W. & FARNHAM, P.J. (1992). The HIP1 binding site is required for growth regulation of the dihydrofolate reductase gene promoter. *Mol. Cell Biol.*, **12**, 1054–1063.

MIHARA, K., CAO, X., YEN, A., CHANDLER, S., DRISCOLL, B., MURPHREE, A.L., T'ANG, A. & FUNG, Y. (1989). Cell cycle-dependent regulation of phosphorylation of the human retinoblastoma gene product. *Science*, **246**, 1300–1303.

MOBERG, K.H., LOGAN, T.J. & TYNDALL, D.J. (1992). Three distinct elements within the murine c-*myc* promoter are required for transcription. *Oncogene*, **7**, 411–421.

MUELLER, C., GRAESMANN, A., & GRAESSMANN, M. (1978). Mapping of early SV40-specific functions by microinjection of different early viral DNA fragments. *Cell*, **15**, 579–585.

QIN, X.-Q., CHITTENDEN, T., LIVINGSTON, D.M. & KAELIN, W.G. (1992). Identification of a growth suppression domain within the retinoblastoma gene product. *Genes & Dev.*, **6**, 953–964.

SOPRANO, K.J., GALANTI, N., JONAK, G.J., MCKERCHER, S., PIPAS, J.M., PEDEN, W.C. & BASERGA, R. (1983). Mutational analysis of simian virus 40 T antigen: stimulation of cellular DNA synthesis and activation of rRNA genes by mutants with deletions in the T-antigen gene. *Mol. Cell Biol.*, **3**, 214–219.

TAKAHASHI, R., HASHIMOTO, T., XU, H.-J., HU, S.-X., MAATSUI, T., MIKI, T., BIGO-MARSHALL, H., AARONSON, S.A. & BENEDICT, W.F. (1991). The retinoblastoma gene functions as a growth and tumor suppressor in human bladder carcinoma cells. *Proc. Natl Acad. Sci. USA*, **88**, 5257–5261.

THALMEIER, K., SYNOVZIK, H., MERTZ, R., WINNACKER, E.-L. & LIPP, M. (1989). Nuclear factor E2F mediates basic transcription and trans-activation by E1A of the human MYC promoter. *Genes & Dev.*, **3**, 527–536.

WHYTE, P., BUCHKOVICH, K.J., HOROWITZ, J.M., FRIEND, S.H., RAYBUCK, M., WEINBERG, R.A. & HARLOW, E. (1988). Association between an oncogene and an antioncogene: the adenovirus E1A proteins bind to the retinoblastoma gene product. *Nature*, **334**, 124–129.

WHYTE, P., WILLIAMSON, N.M. & HARLOW, E. (1989). Cellular targets for transformation by the adenovirus E1A proteins. *Cell*, **56**, 67–75.

XU, H., HU, S., HASHIMOTO, T., TAKAHASHI, R. & BENEDICT, W.F. (1989). The retinoblastoma susceptibility gene product: a characteristic pattern in normal cells and abnormal expression in malignant cells. *Oncogene*, **4**, 807–812.